# Biological Significance of YAP/TAZ in Pancreatic Ductal Adenocarcinoma

**DOI:** 10.3389/fonc.2021.700315

**Published:** 2021-07-29

**Authors:** Hiromitsu Hayashi, Norio Uemura, Liu Zhao, Kazuki Matsumura, Hiroki Sato, Yuta Shiraishi, Hideo Baba

**Affiliations:** Department of Gastroenterological Surgery, Graduate School of Life Sciences, Kumamoto University, Kumamoto, Japan

**Keywords:** pancreatic cancer, Hippo signaling pathway, Yes-associated protein, TAZ, KRAS mutation, pancreatic ductal adenocarcinoma

## Abstract

Pancreatic ductal adenocarcinoma (PDAC) remains one of the most lethal types of cancer. Despite major advances in defining the molecular mutations driving PDAC, this disease remains universally lethal with an overall 5-year survival rate of only about 7–8%. Genetic alterations in PDAC are exemplified by four critical genes (*KRAS*, *TP53*, *CDKN2A*, and *SMAD4*) that are frequently mutated. Among these, *KRAS* mutation ranges from 88% to 100% in several studies. Hippo signaling is an evolutionarily conserved network that plays a key role in normal organ development and tissue regeneration. Its core consists of the serine/threonine kinases mammalian sterile 20-like kinase 1 and 2 (MST1/2) and large tumor suppressor 1 and 2. Interestingly, pancreas-specific *MST1/2* double knockout mice have been reported to display a decreased pancreas mass. Many of the genes involved in the Hippo signaling pathway are recognized as tumor suppressors, while the Hippo transducers Yes-associated protein (YAP) and transcriptional co-activator with PDZ-binding motif (TAZ) are identified as oncogenes. By dephosphorylation, YAP and TAZ accumulate in the nucleus and interact with transcription factors such as TEA domain transcription factor-1, 2, 3, and 4. Dysregulation of Hippo signaling and activation of YAP/TAZ have been recognized in a variety of human solid cancers, including PDAC. Recent studies have elucidated that YAP/TAZ play a crucial role in the induction of acinar-to-ductal metaplasia, an initial step in the progression to PDAC, in genetically engineered mouse models. YAP and TAZ also play a key role in the development of PDAC by both KRAS-dependent and KRAS-independent bypass mechanisms. YAP/TAZ have become extensively studied in PDAC and their biological importance during the development and progression of PDAC has been uncovered. In this review, we summarize the biological significance of a dysregulated Hippo signaling pathway or activated YAP/TAZ in PDAC and propose a role for YAP/TAZ as a therapeutic target.

## Introduction

Pancreatic ductal adenocarcinoma (PDAC) remains one of the most lethal types of cancer (1). Genetic alterations in PDAC are exemplified by four critical genes that are frequently mutated (*KRAS*, *TP53*, *CDKN2A*, and *SMAD4*). Some of these mutations occur when the tumors are in a preneoplastic condition (2). Despite major advances in defining the molecular mutations driving PDAC, this disease remains universally lethal, with an overall 5-year survival rate of only about 7–8%. Although recent developments in systemic chemotherapy such as FOLFIRINOX (5-fluorouracil, folinic acid, irinotecan, and oxaliplatin) and GnP (gemcitabine plus nab-paclitaxel) regimens have provided improved survival outcomes of patients with metastatic PDAC (3, 4), chemoresistance to current systemic chemotherapies (FOLFIRINOX and GnP) is a major treatment issue. Furthermore, of the patients who receive surgical treatments, 60% relapse within 12 months; this is most likely due to micro-metastases that were not detected during the diagnostic computed tomography scan (5). Although approximately 25–30% of patients respond to chemotherapeutic drugs, most eventually become resistant. Resistance mechanisms include deficiencies in drug uptake, alteration of drug targets, activation of DNA repair pathways, and resistance to apoptosis (6). Heterogeneity caused by admixture of tumor cells and stromal cells also produces chemoresistance and limits the targeted therapy of PDAC (7). Unfortunately, our knowledge of the genetic and biological backgrounds in this deadly disease has not yet been linked to improved patient survival. Further developments in therapeutic approaches by continued elucidation of the genetics and molecular biology of PDACs may be the next approach to overcoming this poor prognostic disease and improving survival outcomes.

**Figure 1:**
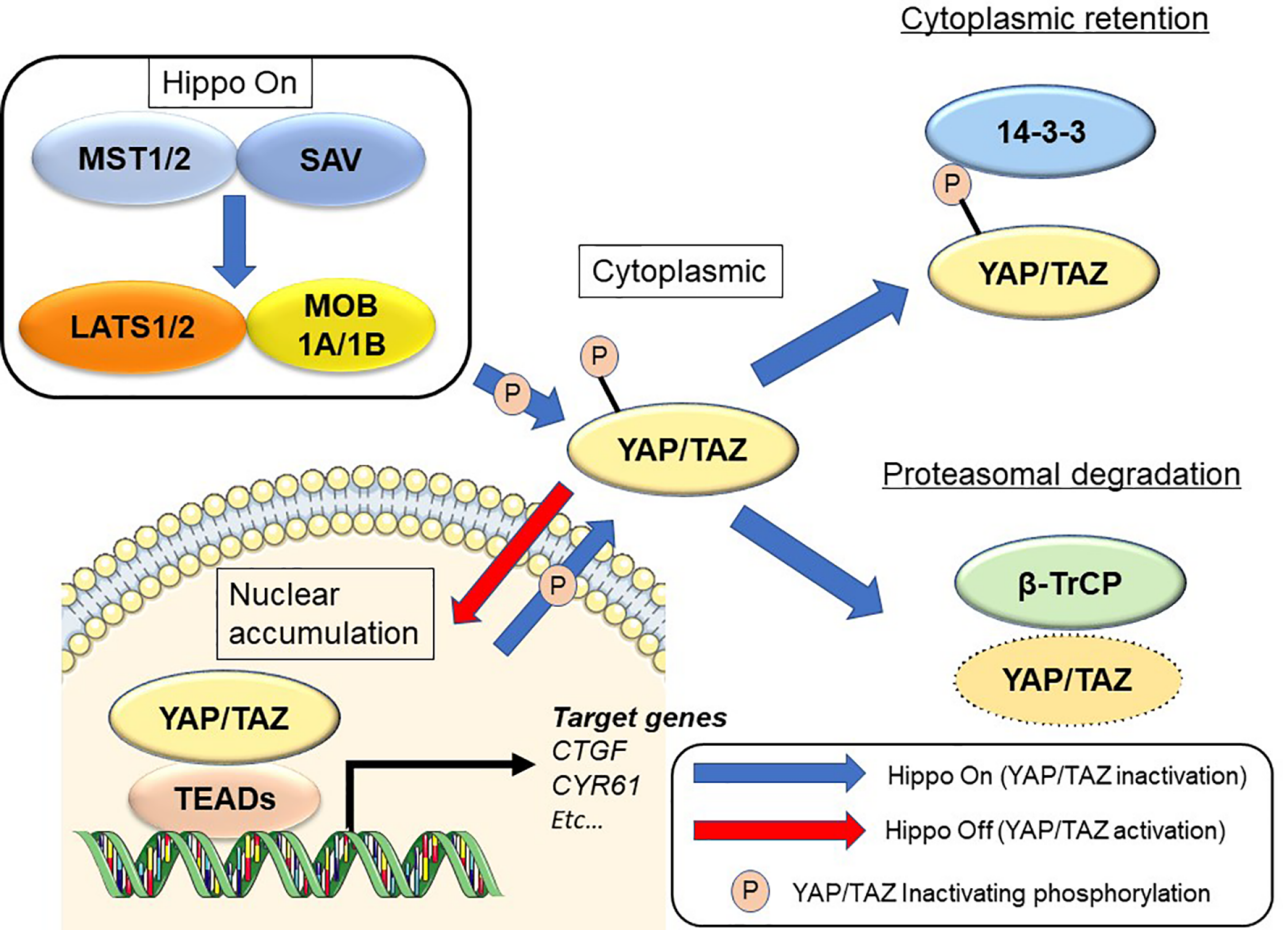
Regulation of the Hippo signaling pathway in mammalian cells. See text for details.

The Hippo signaling pathway was first discovered from studies in *Drosophila melanogaster* (8–10). Hippo signaling is an evolutionarily conserved network that plays a key role in normal organ development and tissue regeneration (11). Multiple inputs control Hippo signaling, ranging from mechanical cues instructed by the cellular microenvironment (mechano-transduction) to soluble factors and metabolic pathways (12, 13). The Hippo pathway also displays extensive crosstalk with other signaling pathways such as transforming growth factor-beta (14, 15), Wnt (16, 17), Sonic hedgehog (18, 19), and Notch (20, 21). Its core consists of the serine/threonine kinases mammalian sterile 20-like kinase 1 and 2 (MST1 and MST2; Hippo in Drosophila) and large tumor suppressor 1 and 2 (LATS1 and LATS2). MST1/2 cooperate with salvador homolog 1 to phosphorylate and activate LATS1/2 kinases. LATS1/2 kinases then combine with the adaptor MOB kinase activator 1 to phosphorylate the Hippo transducers Yes-associated protein (YAP) and transcriptional co-activator with PDZ-binding motif (TAZ) (9, 22) (**Figure 1**). Many of the genes involved in the Hippo signaling pathway are recognized as tumor suppressors, while YAP/TAZ are oncogenes. In addition, YAP and TAZ can be phosphorylated at numerous sites (23, 24). Active LATS1/2 kinases phosphorylate YAP at 5 serine residues (S61, S109, S127, S164, and S381) and TAZ at 4 serine residues (S66, S89, S117, and S311) (23, 24). Among these, S127 (S89 in TAZ; as noted below, the two proteins share moderate sequence similarity) and S381 (S311 in TAZ) are key phosphorylation sites in suppressing YAP/TAZ oncogenic activity (24, 25). Phosphorylation of YAP and TAZ results in their cytoplasmic translocation, sequestration by 14-3-3 proteins, and recruitment of the β-TrCP (SCF) ubiquitin ligase complex (24).

Upon dephosphorylation, YAP and TAZ accumulate in the nucleus and interact with transcription factors such as TEA domain transcriptional factor (TEAD)1, TEAD2, TEAD3, and TEAD4. YAP/TAZ also transcriptionally activate target genes such as connective tissue growth factor (*CTGF*) and cysteine-rich angiogenic inducer 61 (*CYR61*) (11). Deregulation of Hippo signaling has been recognized in a variety of human solid cancers, including PDAC (26–28). YAP/TAZ induce the epithelial-to-mesenchymal transition (EMT) and also induce a more undifferentiated state with malignant behavior in cancer cells (25, 29). YAP/TAZ also contribute to the strongly immunosuppressive microenvironment characteristic of mouse and human PDAC (30). Although YAP and TAZ have very similar structural topologies, share nearly half of their overall amino acid sequences, and are thought to be largely redundant, they may differ in their regulation and downstream functions (31, 32).

YAP/TAZ have become extensively studied in PDAC and their biological importance during the development and progression of PDAC has been uncovered. In this review, we summarize the biological significance of a dysregulated Hippo signaling pathway and activated YAP/TAZ in PDAC, and propose a role for YAP/TAZ as a therapeutic target.

## Biological Role of Hippo Signaling Pathway During Normal Pancreas Development

**Figure 2:**
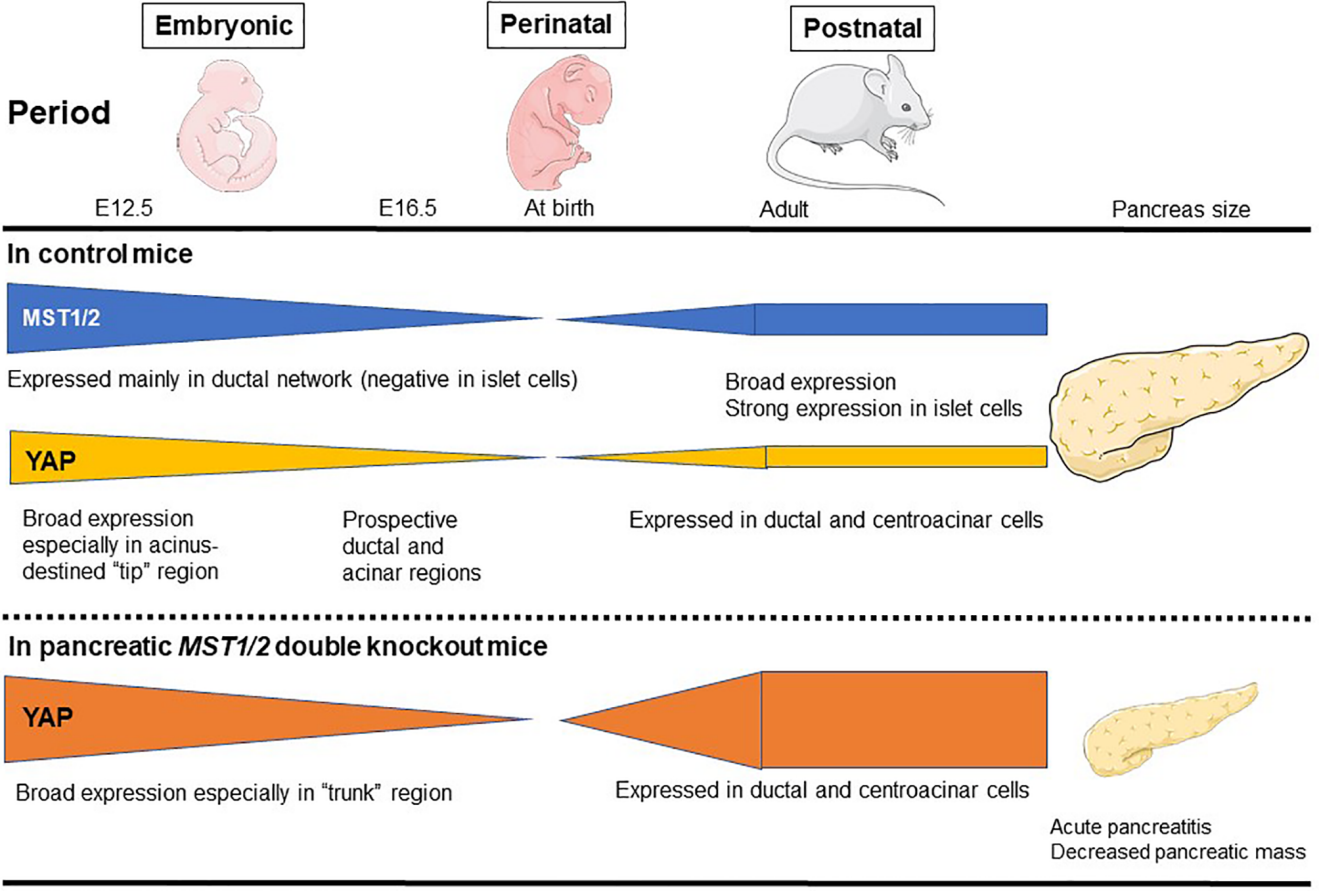
MST1/2 and YAP expression during normal pancreas development. *MST1/2* mRNA levels are highest during the embryonic phase and lowest at birth. *MST1* mRNA expression reappears at postnatal day 7 and later stages. Phosphorylated MST1/2 expression (active Hippo signaling) is broadly detectable in adult human pancreas, and islet cells display strong expression of phosphorylated MST1/2. YAP is broadly expressed throughout the pancreatic and duodenal E12.5 mouse pancreas (primary transition), but then gradually becomes restricted to prospective ductal and acinar regions at E16.5 (secondary transition). In the adult mouse pancreas, YAP expression is markedly decreased and the strong expression is largely confined to ductal and terminal-duct centroacinar cells, unlike in the embryonic pancreas. In pancreatic *MST1/2* double knockout (DKO) mice, nuclear YAP staining is observed in the “trunk” regions during the embryonic phase, but is almost undetectable at birth. Thereafter, YAP expression is weak and confined mainly to the ductal compartment at birth and later postnatal stages. In pancreatic *MST1/2* DKO mice, abundant YAP expression was observed in abnormally numerous duct-like structures during the postnatal phase. YAP remains undetectable within endocrine cells even in the absence of *MST1/2*. Pancreatic *MST1/2* DKO mice show the histologic features of acute pancreatitis and decreased size (an approximately 2-fold decrease in pancreas mass).

The mammalian pancreas is a dual-function organ that is critical for the regulation of basic metabolism. In the mouse, development of the pancreas is divided into two stages, commonly denoted as the primary and secondary transitions (33). In a report using pancreatic *MST1/2* double knockout (DKO) mice, George et al. (34) found that YAP is broadly expressed throughout the pancreatic and duodenal homeobox 1 (Pdx1)-positive embryonic day (E)12.5 mouse pancreas (primary transition) (34) (**Figure 2**). YAP expression then gradually becomes limited to prospective ductal and acinar regions at E16.5 (secondary transition). At E16.5, the productal cells show high YAP expression in the nucleus, whereas acinus-fated cells display expression mainly within the cytoplasm. Strikingly, prospective endocrine cells are negative for YAP expression. Pancreas development at E12.5 is characterized by compartmentalization, whereas E16.5 is characterized by massive cell proliferation and differentiation throughout the pancreas epithelium. In the adult mouse pancreas at 6 weeks (34), YAP expression is markedly decreased and strong expression is largely confined to ductal and terminal-duct centroacinar cells, unlike in the embryonic pancreas. YAP expression in acinar cells displays a weak cytoplasmic staining pattern, and is undetectable within islets. YAP expression in the adult human pancreas mirrors that in the mouse. On the other hand, phosphorylated-MST1/2 expression (indicative of active Hippo signaling) is broadly detectable in adult human pancreas, and islet cells display strong expression of phosphorylated MST1/2 (34). In another report using pancreatic *MST1/2* DKO mice by Gao et al. (35), nuclear YAP staining was observed in the “trunk” regions at E15.5, and was almost undetectable at birth. Thereafter, YAP expression is weak and confined mainly to the ductal compartment at postnatal day (P)7 and later stages. *MST1*/*2* mRNA levels are highest at E15.5 and lowest at birth; *MST1* mRNA expression reappears at P7 and later stages. Interestingly, YAP expression is decreased and absent during the late embryonic and perinatal periods, raising the possibility that YAP must be silenced for proper pancreas differentiation. Such sequential changes in YAP expression have a crucial role for proper pancreas development (34, 35).

Indeed, in pancreatic *MST1/2* DKO mice, abundant YAP expression was observed in the abnormally numerous duct-like structures from P7 to P14 (35). Furthermore, the duct-like cells in *MST1/2* DKO mice originated from acinar cells. In the absence of MST1/2, acinar cells differentiate normally but fail to maintain their differentiated state and de-differentiate or trans-differentiate into a duct-like state (35). On the other hand, *MST1/2* deletion does not affect perinatal YAP expression, suggesting that perinatal YAP repression occurs *via* an MST1/2-independent mechanism (35).

In addition, pancreatic *MST1/2* DKO mice show the histologic features of acute pancreatitis. While no discernible difference is observed between control and pancreatic *Mst1/2* DKO mice at E12.5 (primary transition), a dramatic reduction in the overall expression of amylase is found in the pancreatic *MST1/2* DKO mice at E16.5 (secondary transition), suggesting a defect in exocrine differentiation (34). In these mice, acini fail to form the classic rosette-like structure (34, 35). Robust immune cell infiltration and TUNEL-positive cell death are also detectable with a pancreatitis-like phenotype (35). These findings further suggest that Hippo signaling becomes active during the secondary transition where it regulates acinar cell proliferation and differentiation.

By 6 weeks, the majority of pancreatic cells are not proliferating in mice (34). In contrast, one-third of amylase-positive acinar cells and cytokeratin 19-positive ductal cells display sustained cell proliferation with BrdU incorporation in the pancreatic *MST1/2* DKO mice (34). Thus, MST1/2 play a role as suppressors of proliferation in the mammalian pancreas.

On the other hand, for endocrine cells, YAP is not expressed in glucagon- or insulin-expressing cells at E12.5 and E16.5, respectively (34). Even at P30, YAP is not detectable in the β-cells (35). YAP remains undetectable within endocrine cells even in the absence of MST1/2 (34). Additionally, islet cells are largely Ki-67-negative, in agreement with undetectable YAP expression in both control and pancreatic *MST1/2* DKO mice (34). The ratio of insulin-positive to glucagon-positive cells is not different between control and *MST1/2* DKO mice (34), and blood glucose level also shows no significant difference between them (34, 35). On the other hand, complete loss of YAP in *Yap^flox/flox^:p48*-*Cre* mice also has no effect on blood glucose level (36). Hippo signaling does not play a crucial role in the pancreatic endocrine compartment.

As a consequence of the above features, the pancreas in pancreatic *MST1/2* DKO mice is smaller (approximately 2-fold decrease in pancreas mass), displaying a pale white color and atrophy (34, 35). While Hippo deficiency in liver results in liver hypertrophy (37, 38), the Hippo-deficient pancreas is reduced in size (34, 35). Thus, pancreas mass and tissue architecture are greatly disrupted in the absence of MST1/2. YAP plays a crucial role downstream of MST1/2 during pancreas development, and dysregulation of Hippo signaling may contribute to human pancreatic disease phenotypes.

## Biological Role of the Hippo Signaling Pathway in Pancreatic Cancer Development—Lessons From Genetically Engineered Mouse Models

**Figure 3:**
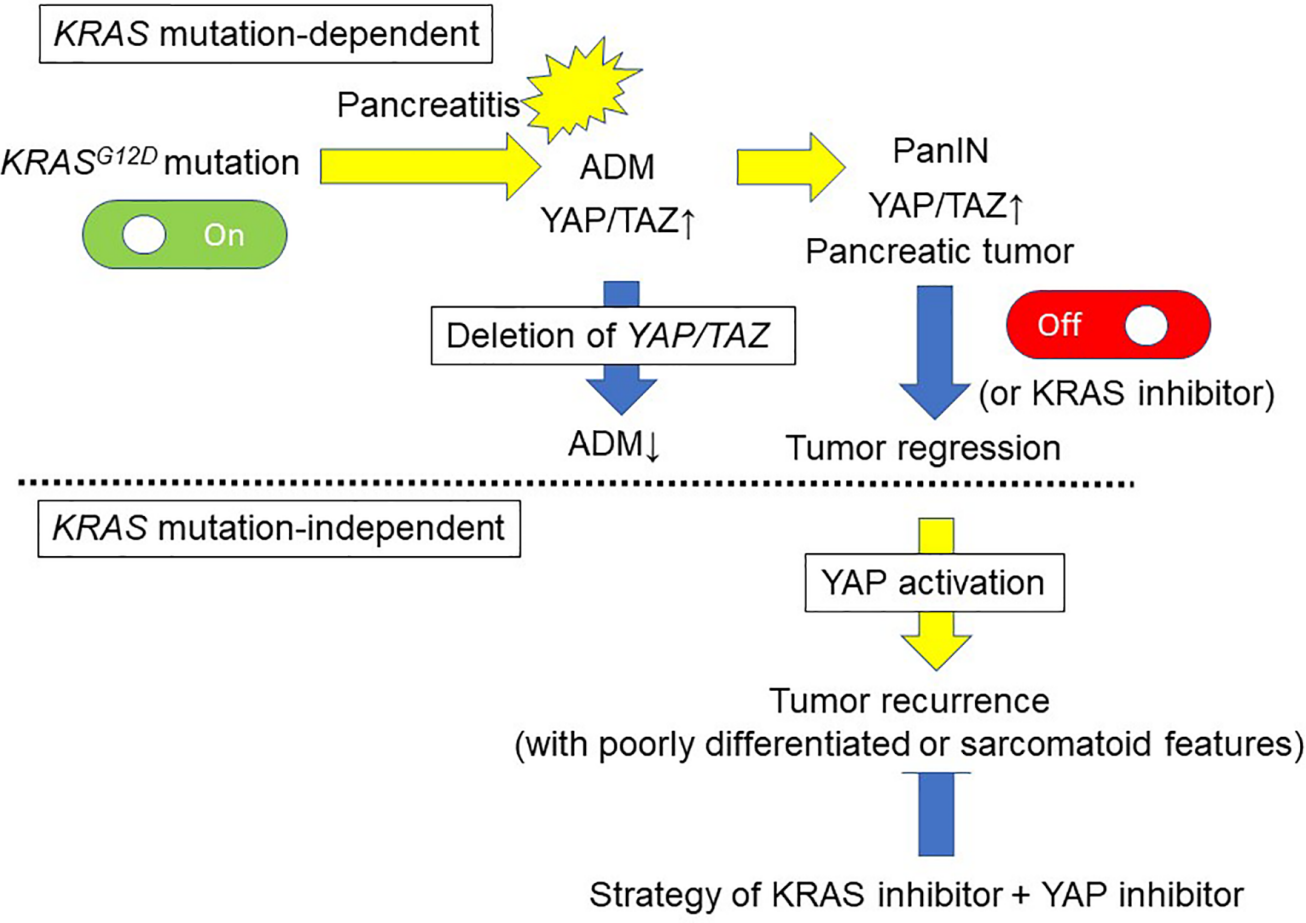
Pancreatic tumor development and maintenance by KRAS-dependent and KRAS-independent mechanisms *via* YAP activation. Oncogenic *KRAS* mutation is a key event of the development of PanIN lesions. Acinar-to-ductal metaplasia (ADM) caused by pancreatitis is an initiating step in pancreatic tumor development. The pancreatic tumor mouse model *LSL-KRAS^G12D^; Pdx1-Cre* displays increased ADM lesions and development of PanIN in response to cerulein, accompanied by YAP/TAZ expression. On the other hand, deletion of YAP/TAZ reduces the ability of *KRAS^G12D^* mutant mice to develop ADM in response to cerulein, and these mice are free of PanIN lesions even after cerulein-induced pancreatitis. Inactivation of *KRAS^G12D^* in established tumor lesions can lead to tumor regression. Although *KRAS^G12D^* extinction induces regression of pancreatic tumors, 70% of the mice develop relapsed tumors *via* oncogenic *KRAS*-independent mechanisms involving the *YAP1* oncogene. An anti-YAP1 therapeutic strategy with KRAS-targeting agents may be required for elective tumors.

The genetic landscape of PDAC is characterized by four frequently mutated genes: *KRAS*, *TP53*, *CDKN2A* (p16), and *SMAD4* (39). The four predominant gene mutations appear to occur sequentially as PanIN progresses. *KRAS* mutations can be found even in normal pancreas and in PanIN1. In PDAC, *KRAS* mutation ranges from 88% to 100% in several studies (40–45). Although the initial step in PDAC development remains to be elucidated, oncogenic *KRAS* mutation is a key event, as evidenced by its presence in PanIN lesions (46, 47) and the development of PanIN lesions in oncogenic *KRAS*-driven genetically engineered mouse models (GEMMs) (48, 49). The oncogenic *KRAS* mutation perturbs the constitutively activated RAS protein, and results in the dysregulated activation of proliferation and survival pathways. GEMMs have provided several insights into the development of PDAC (50–53). Although oncogenic *KRAS* mutations are recognized early events in PDAC development, they are not entirely sufficient for the development of fully invasive PDAC. Indeed, only 5–10% of animals in GEMMs with mutated *KRAS* (without additional genetic alterations) develop frank PDAC, and do so very late (usually after 9 months) (50). PDAC development can be enhanced by the existence of another mutation (e.g., *TP53*) (51, 54). Pancreatic inflammation by administration of cerulein accelerates the formation of PanINs and PDAC in *KRAS^G12V^* mice (55). In addition to the role of oncogenic KRAS in development of PDAC, *KRAS* mutations have also been shown to be important for PDAC maintenance (56, 57). Interestingly, inactivation of *KRAS^G12D^* in confirmed precursor lesions and during progression to PDAC leads to tumor regression of those lesions, showing that *KRAS^G12D^* is required for tumor cell maintenance (56, 58) (**Figure 3**). In an analysis of *KRAS* mutation type, codon G12D mutation was the most frequent (48%), followed by G12V (31%) and G12R (21%) (45).

Acinar-to-ductal metaplasia (ADM) caused by pancreatitis is an initiating step in pancreatic tumor development (55). Cerulein treatment reduces phosphorylation of LATS1, and increases YAP/TAZ protein levels accompanied by strong nuclear localization (59). Following cerulein treatment, cytokeratin 19 (duct cell marker) expression is also increased, consistent with acinar-to-ductal reprogramming (59). Thus, YAP/TAZ activity is accelerated in the injured pancreas, particularly in the subset of cells undergoing ADM (**Figure 3**).

Recent studies have demonstrated that YAP/TAZ play a crucial role in the induction of ADM, an initial step in the progression to PDAC, in GEMMs (36, 59). The pancreatic tumor mouse model *LSL-KRAS^G12D^;Pdx1-Cre* displays the whole spectrum of preneoplastic lesions (50). In these mice, increased ADM lesions and development of PanIN with strong YAP/TAZ expression are detectable, and YAP/TAZ levels are elevated in pancreatic protein lysates (59). Deletion of *YAP/TAZ* significantly reduced the ability of *KRAS^G12D^* mice to induce ADM in response to cerulein, and these mice (*KRAS^G12D^;YAP1^fl/fl^;TAZ^fl/fl^*) were free of PanIN lesions at 3 months after the transient induction of pancreatitis by cerulein, similar to control mice (59). Thus, YAP/TAZ are required for *KRAS^G12D^*-induced ADM in response to pancreatitis *in vivo* (**Figure 3**). Deletion of *YAP/TAZ* in the *KRAS^G12D^* mice reduced Ras activation even after cerulein treatment (59). In contrast, ectopic YAP/TAZ activation in acinar cells by adenoviral vectors converted the infected acinar cells to duct cell morphology (59). Overexpression of constitutively active YAP1 in primary acinar cells also enhances Ras activity (59). YAP/TAZ are necessary and sufficient for ADM induction (59). Acinar cell-specific YAP/TAZ signaling may be essential for oncogenic *KRAS^G12D^*-induced PanIN formation in the context of pancreatitis.

Zhang et al. (36) genetically engineered *KRAS^G12D/+^:TP53^R172H/+^:YAP^flox/flox^:p48-Cre* mice to determine whether YAP is involved in PDAC development. In their study, *KRAS^G12D/+^:p48-Cre* or *KRAS^G12D/+^:TP53^R172H/+^:p48-Cre* mice with one or two intact *YAP* alleles developed ADM and early PanINs from 4 to 8 weeks of age, respectively (36). These ADM and early PanINs progressed through late-stage PanINs and eventually to invasive PDAC by 2 to 4 months in *KRAS^G12D/+^:TP53^R172H/+^:p48-Cre* mice, or from 6 months to 2 years in *KRAS^G12D/+^:p48-Cre* or *KRAS^G12D/+^* mice (36). In contrast, when these mice underwent homozygous *YAP* deletion (*KRAS^G12D/+^:YAP^flox/flox^:p48-Cre* and *KRAS^G12D/+^:TP53^R172H/+^:YAP^flox/flox^:p48-Cre*), they entirely lacked any late-stage PanINs or PDAC (36).

Zhang et al. generated *p48-Cre*;*LSL-KRAS^G12D^;FBXW7^fl/fl^* mice to examine whether loss of the tumor suppressor FBXW7 might be an additional gene alteration in the development of PDAC (60). They found that the mice displayed accelerated tumorigenesis: PDACs were detectable by P14 and all mice yielded PDACs by P40 PDAC in *p48-Cre*;*LSL-KRAS^G12D^;FBXW7^fl/fl^* mice was preceded by earlier onset of ADM and PanIN lesions, and accompanied by chromosomal instability and the accumulation of YAP (60). Furthermore, in a pancreatic cell line established from *p48-Cre*;*LSL-KRAS^G12D^;FBXW7^fl/fl^* mice and in *FBXW7*-deficient human pancreatic cancer cells, down-regulation of YAP attenuated cell growth. Thus, deletion of the tumor suppressor FBXW7 accelerates *KRAS*-driven pancreatic tumorigenesis with YAP expression (60).

Kapoor et al. examined the mechanism of *KRAS^G12D^*-independent PDAC recurrence using a doxycycline-inducible *KRAS^G12D^* transgene and conditional *p53* null alleles (*p48Cre; tetO_LSL-KRAS^G12D^; ROSA_rtTA; p53L/+*, designated iKras) (61). In their investigation, *KRAS^G12D^* extinction by doxycycline withdrawal induced complete regression of pancreatic tumors at three weeks, as determined by MRI imaging. However, 70% of the mice developed relapsed tumors between 9 and 47 weeks after doxycycline withdrawal, with a median survival of 36.6 weeks compared to 15.4 weeks for iKras mice with continued doxycycline treatment (61). Kapoor et al. revealed oncogenic *KRAS*-independent bypass mechanisms involving the *YAP1* oncogene in *KRAS^G12D^*-independent PDAC recurrence, and emphasized the potential importance of an anti-YAP1 therapeutic strategy for elective tumors in the clinical setting with agents that targeted KRAS and its signaling pathways (61) (**Figure 2**). Shao et al. reported similar mechanisms in *KRAS*-driven lung cancer (62). These findings suggest that murine PDAC cells can survive in the absence of oncogenic *KRAS* signaling and acquire alternative mechanisms to foster their own growth (61, 63). The activity of the transcriptional co-activator YAP plays a critical role in the promotion and maintenance of PDAC by operating as a key downstream target of KRAS signaling. *YAP/TAZ* amplification frequency ranged from 0% to 19% in 9,125 tumor samples among 33 cancer types from The Cancer Genome Atlas (64). Among these 33, the top six cancer types with the highest amplification frequency of *YAP/TAZ* included all five squamous cell-involved cancers (lung squamous cell carcinoma, esophageal squamous cell carcinoma, head and neck squamous cell carcinomas, and bladder urothelial carcinoma), whereas the frequency in PDAC was about 2% and 14th among 33 cancer types (64). Overall, YAP has emerged as a central node of transcriptional convergence in growth-promoting signaling in PDAC cells by both KRAS-dependent and KRAS-independent bypass mechanisms. (**Figure 3**). Collectively, these observations indicate that YAP/TAZ also play a crucial role in the development and recurrence of PDACs.

## Clinical Impact of YAP/TAZ Expression in PDAC Patients

The clinical function of YAP as a prognostic marker has been investigated in several studies (**Table 1**), which have indicated that YAP and/or TAZ are overexpressed in tumor samples from patients with PDACs (61, 65–68). It has been found that nuclear overexpression of YAP is an independent prognostic marker for poor survival and is associated with liver metastasis (68). Furthermore, using public mRNA expression data, YAP was confirmed to be correlated with poor survival (69, 70). The 5-year survival rate was 0% in patients with high *YAP* mRNA expression compared to 32% in those with low expression. Furthermore, multiple YAP/TEAD-regulated genes were associated with poor prognosis, such as transforming growth factor alpha, heparin-binding EGF-like growth factor, integrin subunit alpha 2, P2Y2 receptor, G protein-coupled receptor 87, and mucin 1. On the other hand, YAP-inhibitory pathway components were associated with a favorable prognosis, such as STE20-related kinase adaptor/liver kinase B1, protein kinase A/large tumor suppressor, and tuberous sclerosis complex/mammalian target of rapamycin complex 1.

**Table 1:** YAP/TAZ expression and functional relevance in human pancreatic cancers.

Reference	Number	Target	Location	Positive ratio	Outcomes
Diep et al. (65)					
	64	YAP1	Primary	77%	Not available
Yang et al. (66)					
	38	YAP	Primary	61%	Not available
	25	YAP	Metastatic site	72%	Not available
Xie et al. (67)					
	57	TAZ	Primary	82%*	Not available
Salcedo Allende et al. (68)					
	64	YAP1	Primary	90.62%	Poor OS
Rozengurt et al. (69)**					
	176	*YAP* mRNA	Primary	20%	Poor OS
Zhou et al. (70)***					
	176	*YAP1* mRNA	Primary	50%	Poor OS

*Weak, moderate, and strong expression of TAZ were identified as positive.

**A published interactive open-access database (www.proteinatlas.org/pathology) was used.

***The cohort of The Cancer Genome Atlas (TCGA) was used.

OS, overall survival.

## Biological Role of YAP/TAZ in Cancer Cells

There is accumulating evidence that YAP and TAZ promote proliferation and growth of PDAC cells. Treatment of PDAC cells with YAP-targeting small interfering RNA oligonucleotides significantly reduced tumor growth (65). It has been reported that eukaryotic translation initiation factor 5A–pseudopodium-enriched atypical kinase 1 signaling regulates YAP and TAZ expression and pancreatic cancer cell growth (71). Disrupting this signaling in pancreatic cancer cells inhibited YAP/TAZ protein expression, reducing the expression of stem cell-associated transcription factors and tumor sphere growth (71).

In human PDAC cells, YAP functions as a downstream effector of the crosstalk between insulin/IGF-1 receptor and G protein-coupled receptor systems (72). Stimulation with insulin and the G protein-coupled receptor agonist neurotensin induced rapid YAP nuclear import and markedly augmented the mRNA levels of YAP/TEAD-regulated genes, including *CTGF* and *Cyr61*. The growth-promoting agonists regulated YAP activity *via* PI3K and protein kinase D in PANC-1 and MiaPaCa-2 (72), human cell lines that correspond to the squamous/quasi mesenchymal/basal-like sub-type of PDAC. It is of great interest that YAP function has been associated with this PDAC sub-type, considered the most clinically aggressive form.

The epithelial-to-mesenchymal transition (EMT) is a developmental regulatory program defined by the phenotypical transition from an epithelial to a mesenchymal cell state. The EMT is an essential step for metastasis and confers resistance to therapy (73). Active YAP promotes pancreatic cancer cell motility, invasion, and tumorigenesis in a mitotic phosphorylation-dependent manner and contributes to the EMT in pancreatic cancer cells by several mechanisms, including hyperactivation of AKT signaling (66, 67, 74, 75). YAP/TAZ also interact with nuclear factors such as ZEB1 (29) and SMADs (76, 77), both of which are important EMT regulators. TGF-β is a well-known EMT inducer in cancer cells. TGF-β enhances YAP nuclear localization and stabilizes YAP activity, and TGF-β-induced EMT and YAP activity are both blocked by inhibition of AKT signaling in PDAC cells (78). Xie et al. (67) focused on TAZ activation in pancreatic cancer cells and examined its functional roles in the EMT. Aberrant expression and activation of TAZ in pancreatic cancer cells promoted the EMT *via* down-regulation of E-cadherin and up-regulation of vimentin expression. In contrast, depletion of *TAZ* in pancreatic cancer cells suppressed the EMT phenotype (67).

PDAC is characterized by a high degree of chemoresistance. Gemcitabine has been the standard chemotherapeutic agent in PDAC since 1997 (79). Several mechanisms of YAP-induced chemoresistance have been proposed. One mechanism suggests that YAP overexpression induces the EMT in pancreatic cancer cells by activating the AKT cascade, which can cause resistance to gemcitabine (74). Another proposed mechanism involves microRNA, since microRNA 181c was overexpressed in PDAC samples and correlated with poor prognosis. microRNA 181c directly repressed MST1, LATS2, salvador homolog 1, and MOB kinase activator 1, leading to YAP and TAZ activation, and gemcitabine resistance *in vitro* and *in vivo* (80). Isoprenylcysteine carboxylmethyltransferase (ICMT) is the catalytic enzymes in the three step prenylation processing that posttranslationally modifies substrate proteins including RAS isoforms. Suppression of ICMT inhibits cancer stem cell self-renewal and chemoresistance of mutant KRAS pancreatic cancer cells with TAZ protein degradation (81). On the other hand, expression of constitutively active KRAS*^G12V^* restores TAZ protein level and the self-renewal ability of pancreatic cancer cells. Thus, mutant KRAS plays a major role in TAZ expression and cancer stem self-renewal in pancreatic cancer cells, and ICMT has potential as a pharmacological target in the treatment of mutant KRAS pancreatic cancer cells (81).

## Biological Role of YAP/TAZ in the Tumor Microenvironment

An important feature of human and murine PDAC is an extensive desmoplastic stroma (82) that increases the stiffness of the extracellular matrix (ECM) surrounding epithelial cancer cells (83). The Hippo/YAP pathway has been recognized to play a critical role in mechano-transduction (84, 85) and in sensing ECM stiffness (86). High stiffness leads to inhibition of the Hippo tumor suppressive pathway while enhancing the activity of YAP/TAZ. The stroma contains cancer-associated fibroblasts (CAFs), immune cells, endothelial cells, and the ECM. Pancreatic stellate cells are resident mesenchymal cells of the pancreas that represent the major source of CAFs. It has been found that YAP and TAZ are expressed at high levels in activated pancreatic stellate cells in PDAC, as well as in chronic pancreatitis (87).

Transglutaminase 2 secreted by pancreatic cancer cells promotes cross-linking of collagen, which activates CAFs and stimulates their proliferation, and results in higher collagen production by CAFs and further stiffening of the stroma. In turn, such a stiff tumor microenvironment conveys mechanical signals to cancer cells, leading to activation of YAP/TAZ and tumor progression (88). Environmental stimuli, including obesity and metabolic syndrome, also enhance the promotion of invasive PDAC (89, 90).

PDAC is characterized by a profound inflammatory reaction and an immunosuppressive state (91). Pancreatic tumors are associated with immune dysfunction, partly mediated by the recruitment of immunosuppressive cells, such as tumor-associated macrophages and myeloid-derived suppressor cells (92, 93). These cells are recruited to the tumor microenvironment and can inhibit T-cell activity. YAP has been identified as a critical regulator of the immunosuppressive microenvironment in PDAC. YAP inactivation prevented recruitment of myeloid-derived suppressor cells while in turn supporting infiltration of antigen-presenting macrophages and T-cell activation, thereby promoting apoptosis of tumor cells (30). Although T-cell activity is critical for tumor immunity, T-cell fate is governed by Hippo signaling (94–96). Geng et al. reported that TAZ induces Th17 cell differentiation and suppresses the differentiation of immunosuppressive regulatory T-cells (95). Ni et al. reported that immunosuppressive activity of regulatory T-cells was dependent on YAP expression in melanoma, and the anti-tumor immunity was enhanced in the absence of YAP (97). In hepatocellular carcinoma, YAP mediates the migration of macrophages *in vitro* and *in vivo* (98). Thus, YAP/TAZ are capable of regulating the biological activity and function of T-cells and macrophages, which is crucial for tumor immunity. They thereby participate in immune escape by suppressing normal immunological activity.

Whole-genome and whole-exome sequencing of PDACs have revealed a mean mutation load of 1.8 and 1.1 mutations per megabase, respectively, and only 5% of PDACs displayed a hypermutated phenotype (99). The identification of hypermutated PDACs is important because these tumors are sensitive to immunotherapy (99). Furthermore, the prevalence of microsatellite instability was found to be around 5% in many solid tumors, while in PDAC it was only 2% (100). These DNA mismatch repair (MMR)-deficient tumors carried high neo-antigen load and displayed considerably improved responses to programmed cell death 1 blockade (100). These authors reported that solid tumors with MMR deficiency are responsive to immune checkpoint blockade with pembrolizumab. Pembrolizumab has subsequently been approved by the FDA for solid tumors with MMR deficiency, regardless of tissue of origin (101). Furthermore, the clinical benefit of pembrolizumab was confirmed in patients with microsatellite instability high MMR-deficient non-colorectal cancers including pancreatic cancer (102). Thus, immunotherapy is a rapidly progressing field in cancer treatment. Among the immunotherapy modalities, immune checkpoint inhibition has displayed considerable success in several solid tumors, but there is still no significant benefit in PDAC.

In cancers other than PDAC, there is accumulating evidence that YAP/TAZ play a pivotal role in PD-L1 expression. Overexpression of constitutively active YAP or TAZ by the deletion of MST1/2 or LATS1/2 enhances PD-L1 expression in breast and lung cancer cell lines (103). Furthermore, PD-L1 expression is also induced by YAP in BRAF inhibitor-resistant melanoma, and the relationship between YAP and PD-L1 expression was validated in human clinical melanoma tissues (104). In human non-small cell lung cancer, YAP regulated PD-L1 at the transcriptional level, suggesting that YAP has potential as an immunotherapeutic target (105). Lee et al. found that YAP regulates PD-L1 by directly binding to the PD-L1 promoter and that YAP/PD-L1 signaling modulated tumor cell proliferation and migration in EGFR–TKI-resistant lung adenocarcinoma, and also that YAP down-regulation inhibited PD-L1 expression (106). It is worth further exploring the role of YAP/TAZ in tumor immunotherapy. Thus, targeting YAP/TAZ may be an alternative approach for combination with immunotherapy in cancer cells and the tumor microenvironment.

## Therapeutic Targeting of the Hippo Signaling Pathway in PDAC

According to the above collected evidence, it is reasonable to develop drugs that target YAP and TAZ activities in PDAC. As indicated above, tumor cells with YAP activation can evade the requirement for *KRAS* mutant expression in PDAC (63). YAP is a key element not only downstream of Ras but also as an alternative route to bypass the need of this oncogene for tumor relapse. Recently, the KRAS^G12C^ inhibitor Sotorasib is effectively developed for solid cancers (107). In 11 PDAC patients with *KRAS^G12C^* mutation, one patient had a confirmed partial response, 9 had stable disease, and one had progressive disease in response to Sotorasib (107). Even if Ras could be effectively inhibited by this new therapy, YAP amplification offers a potential pathway to induce tumor recurrence. Recent studies suggest novel approaches to inhibit YAP/TAZ activity with drug repositioning in clinical use, including statins. Statins, which have been used to treat dyslipidemia and prevent heart diseases, selectively inhibit 3-hydroxy-methylglutaryl (HMG) CoA reductase (108), the rate-limiting enzyme in the generation of mevalonate. Accelerated mevalonate biosynthesis through mutant *p53* (109–111) and AKT/mTORC1 (111) has been reported in cancers. The mevalonate pathway plays an important role in the generation of lipids and lipid intermediates, including farnesyl pyrophosphate, geranylgeranyl pyrophosphate, and cholesterol. In preclinical studies (112, 113), statins delayed the progression of PDAC in mice harboring *KRAS^G12D^* mutation. Statins were identified as potential YAP inhibitors by screens of molecules that changed the nuclear/cytoplasmic distribution of YAP (114). In our previous study, statin treatment suppressed cancer cell growth *via* TAZ down-regulation in hepatocellular carcinoma (115). Several epidemiological studies have indicated that statin use correlates with favorable oncologic effects in PDAC (116–124), especially in males (119, 120). A large study demonstrated that statins were associated with a significantly reduced PDAC risk (by 34%), with a stronger effect in males (119). The beneficial effects of statins depend on the type of statins used, with several reports showing positive associations with lipophilic (and not hydrophilic) statins and reduced cancer risk (125–128). On the other hand, Hamada et al. reported that regular statin use was not associated with pancreatic cancer risk in two large prospective cohort studies in the U.S (129). Nevertheless, Hamada et al. also reported increased survival in PDAC patients with regular pre-diagnosis use of statins (130). Recently, a meta-analysis of PDAC risk that included more than 3 million participants and 170,000 PDAC patients has been published (131). This study indicates a significant decrease in PDAC risk with statin use, thus reinforcing the conclusion that statin administration is associated with beneficial effects in PDAC patients. In addition to their potential efficacy in primary prevention and interception, statins may improve the outcome for patients after surgical removal of their primary PDAC (116, 117, 132), indicating a possible role for statins in the prevention of PDAC recurrence. Collectively, accumulating evidence from epidemiological and preclinical studies indicates a protective effect of statins in PDAC. Of the evaluated treatments in PDAC, verteporfin (133, 134) has a direct effect on Hippo signaling by inhibiting YAP–TEAD interactions. Erlotinib (135), FG-3019 (CTGF antagonism) (136), BIS 1 (135), LY3009120 (133) and ICMT small molecule inhibitor (81) indirectly affect YAP and/or TAZ signaling. Although the mechanism is not fully clarified for several natural substances, curcumin (32, 137), resveratrol (138), *Stichopus japonicus* acidic mucopolysaccharide (139), and pseudolaric acid B (140) have been reported to target YAP/TAZ signaling. Also, in our previous study, curcumin, a major component of turmeric and an old Indian spice, successfully suppressed TAZ/YAP expression and exerted anticancer effects in hepatocellular carcinoma cell lines (32). In the future, direct or indirect pharmacological modulation of YAP/TAZ expression may become promising approaches to fight this deadly disease.

## Conclusions

The Hippo pathway is an evolutionarily conserved signaling pathway in mammals, and YAP and TAZ are key downstream regulators in the Hippo pathway that play a crucial role in the development of the normal pancreas and of PDAC in GEMMs. Furthermore, YAP and TAZ play a crucial role in the development of PDAC by both KRAS-dependent and KRAS-independent bypass mechanisms. Also in PDAC progression, aberrant transcriptional activity of YAP and TAZ has a pivotal role in malignant behavior, including cell growth, EMT, and drug resistance. Besides, YAP/TAZ play a tumor-promoting role in the tumor microenvironment. PDAC features an extensive desmoplastic stroma, and the stroma contains CAFs and immune cells. YAP promotes CAF activation and subsequent fibroinflammatory responses, and the resultant high stiffness enhances the malignant behavior of PDAC with high activity of YAP/TAZ. In addition, YAP acts as a critical regulator of the immunosuppressive microenvironment in PDAC. YAP/TAZ have potential as a therapeutic target not only for cancer cells, but also for the tumor microenvironment in PDAC. Thus, accumulating evidence supports the biological importance of YAP/TAZ in the development and progression of PDACs, and the regulation of YAP/TAZ signaling is increasingly recognized as a therapeutic target. In the near future, direct or indirect pharmacological modulation of YAP/TAZ may become promising therapeutic approaches in PDACs. On the other hand, complete deletion of YAP in knockout mice induced embryonic lethality at E8.5 due to severe developmental defects (141). Although TAZ knockout mice show only partial lethality, with 20% of the mice remaining viable, the survivors develop renal cysts and lung emphysema (142–144). Since YAP/TAZ has so many important physiological functions, as evidenced by YAP-null and TAZ-null mice, careful targeting of the YAP/TAZ signaling pathway to minimize systemic effects is clearly a highly desirable goal in PDAC treatment. Anti-YAP/TAZ strategies to selectively block aberrant YAP/TAZ signal activation are attractive and rational. Biomarker analysis to identify aberrant YAP/TAZ signal activation may therefore be the next step to establish an efficient therapeutic approach.

## Author Contributions

HH and HB conducted the topic investigated in this paper. NU, LS, KM, HS, and YS assisted in the useful discussions and wrote the manuscript. All authors contributed to the article and approved the submitted version.

## Funding

This work was supported by a Grant-in-Aid for Scientists (C); the Ministry of Education, Culture, Sports, Science, and Technology of Japan, No. 19K09177 (to HH); the Shinnihon Foundation of Advanced Medical Treatment Research (HH), the Takeda Science Foundation, Japan (to HH), and Public Trust Surgery Research Fund (to HH).

## Conflict of Interest

The authors declare that the research was conducted in the absence of any commercial or financial relationships that could be construed as a potential conflict of interest.

## Publisher’s Note

All claims expressed in this article are solely those of the authors and do not necessarily represent those of their affiliated organizations, or those of the publisher, the editors and the reviewers. Any product that may be evaluated in this article, or claim that may be made by its manufacturer, is not guaranteed or endorsed by the publisher.

## Abbreviations

ADM, Acinar-to-ductal metaplasia; CAFs, cancer-associated fibroblasts; CTGF, connective tissue growth factor, CYR61, cysteine-rich angiogenic inducer 61; DKO, double knockout; ECM, extracellular matrix; EMT, epithelial-to-mesenchymal transition; FBXW7, F-box and WD repeat domain containing 7; FOLFIRINOX, 5-fluorouracil, folinic acid, irinotecan, and oxaliplatin; GEMM, genetically engineered mouse model; GnP, gemcitabine plus nab-paclitaxel; ICMT, Isoprenylcysteine carboxylmethyltransferase; LATS1/2, large tumor suppressor 1/2; MST1/2, mammalian sterile 20-like kinase 1/2; MMR, mismatch repair; PDAC, pancreatic ductal adenocarcinoma; TAZ, transcriptional co-activator with PDZ-binding motif; TEAD, TEA domain transcriptional factor; PD-L1, programmed cell death-ligand 1; Pdx-1, pancreatic and duodenal homeobox 1; YAP, Yes-associated protein.
